# Taxonomic clarifications concerning the crocodyliform genus *Isisfordia*

**DOI:** 10.7717/peerj.8630

**Published:** 2020-02-25

**Authors:** Lachlan J. Hart

**Affiliations:** Palaeoscience Research Centre, School of Environmental and Rural Science, University of New England, Armidale, New South Wales, Australia

**Keywords:** *Isisfordia*, *Selaslophensis*, *Molnari*, *Duncani*, Griman creek, Lightning ridge

## Abstract

**Background:**

In a recent paper, a new species of the crocodyliform genus *Isisfordia* was erected based on, in part, a specimen previously designated as the holotype of *‘Crocodylus (Bottosaurus)’ selaslophensis*. This new species was given the name *Isisfordia molnari*. However, because the holotype of *‘Crocodylus (Bottosaurus)’ selaslophensis* displays a unique combination of characters and does not overlap with the holotype of *I. molnari*, both names remain valid according to ICZN regulations.

**Results:**

The present work instates *Isisfordia selaslophensis* comb. nov., recognising the seniority of the original specific epithet given to the specimen. The specimen is also reaffirmed as the holotype of the species. *Isisfordia molnari* is rediagnosed based on non-overlapping material but is potentially referable to *Isisfordia selaslophensis*. All other analyses, descriptions, diagnoses and conclusions stated by the original study remain valid.

## Introduction

Hart et al. (2019) recently described a new species of the crocodyliform *Isisfordia* from the Cenomanian-aged Griman Creek Formation at Lightning Ridge (northern New South Wales, Australia). The new taxon, *Isisfordia molnari*, was based on the holotypic braincase (AM F125553) and a referred maxillary fragment (AM F15818). The maxillary fragment was previously designated as the holotype of *‘Crocodylus (Bottosaurus)’ selaslophensis* Etheridge, 1917 *emend.* Molnar (1980) but was regarded as a *nomen dubium* by Mannion et al. (2015; supp. info) owing to a lack of “taxonomic opinion data” (pg. 9). As discussed by Hart et al. (2019), AM F15818 does not show any significant similarity to either *Crocodylus* or *Bottosaurus*, yet shares characteristics consistent with *Isisfordia*, namely a caudal maxillary alveolar groove. However, differences in the tooth and alveolar morphology separate AM F15818 from *Isisfordia duncani,* the type, and only other known species of the genus (Hart et al., 2019). Based on this, AM F15818 (together with AM F125553) was considered to represent a distinct species of *Isisfordia.* Hart et al. (2019) gave this new species the name *Isisfordia molnari* and diagnosed in based on features of the holotype (the braincase) and referred specimen (the maxilla). However, because the referred specimen (AM F15818) displays a unique combination of characters (caudal maxillary alveolar groove, labiolingually compressed, lingually curved tooth crowns, thickening of the medial alveolar wall, rounded alveolar shape, and the continuous arrangement of the caudal maxillary alveolar septa), the previously-designated specific epithet has taxonomic seniority. *Isisfordia molnari* remains a valid nomenclatural act under ICZN (International Commission on Zoological Nomenclature) guidelines but is likely synonymous with *I. selaslophensis* comb. nov.

The present work clarifies this taxonomic synonymy and has been registered in ZooBank, thus meeting the ICZN regulations.

## Materials & Methods

The electronic version of this article in Portable Document Format (PDF) will represent a published work according to the International Commission on Zoological Nomenclature (ICZN), and hence the new names contained in the electronic version are effectively published under that Code from the electronic edition alone. This published work and the nomenclatural acts it contains have been registered in ZooBank, the online registration system for the ICZN. The ZooBank LSIDs (Life Science Identifiers) can be resolved and the associated information viewed through any standard web browser by appending the LSID to the prefix http://zoobank.org/. The LSID for this publication is urn:lsid:zoobank.org:pub:0FD2AD7B-F46D-42C6-B925-D09BACFEFFB0. The online version of this work is archived and available from the following digital repositories: PeerJ, PubMed Central and CLOCKSS.

## Systematic Palaeontology

**Table:** 

CROCODYLIFORMES Hay, 1930
MESOEUCROCODYLIA Whetstone and Whybrow, 1983
NEOSUCHIA Clark, 1988
EUSUCHIA Huxley, 1875
Genus ISISFORDIA Salisbury et al., 2006

Diagnosis (autapomorphies marked with an ‘a’): Broad exposure of the prootic within the supratemporal foramen rostral to the rostral aperture of the posttemportal canal (a); caudal maxillary alveolar groove (a); maximum diameter of the caudal aperture of the cranioquadrate siphonium approximately one-third the mediolateral width of the foramen magnum, with the lateral wall of the siphonium formed exclusively by the quadrate (a); maximum mediolateral width of the secondary choanae exceeds the minimum mediolateral width of the palatines; naris with a distinctly pear-shaped outline (a); caudal dentary teeth confluent and set in a shallow alveolar groove (shared with some alligatoroids); dentary and maxillary teeth flattened labiolingually at the base of the crown, but become conical towards the apex; cervical, thoracic and cranial-most caudal vertebrae weakly procoelous at maturity (a); caudal vertebrae weakly procoelous (a); sacral vertebra II with a low caudal condyle (a); distal extremity of ulna expanded transversely with respect to the long axis of the bone (shared with *Susisuchus* spp. and *Theriosuchus pusillus*).

**Table:** 

***Isisfordia duncani*Salisbury et al., 2006**

 Holotype: QM F36211 (near complete skeleton, missing the rostral part of the skull).

Referred material: QM F44320 (skull) QM F44319 (partial maxilla and mandible), QM F34642 (partial articulated skeleton).

Locality, horizon and age: ‘lower’ Winton Formation, uppermost Albian–Cenomanian, Queensland.

Diagnosis: Species of *Isisfordia* with the following autapomorphies: median ridge on parietal; ridges on the parietal forming the medial margin of the supratemporal foramina; caudal maxillary tooth crown bases and alveoli ovate.

**Table:** 

***Isisfordia selaslophensis* (Etheridge, 1917) comb. nov.**

**Taxonomic assessment**

Holotype: AM F15818 (maxillary fragment; holotype of *‘Crocodylus (Bottosaurus)’ selaslophensis*).

Illustrations of material: Hart et al. (2019: figs. 4, 5).

Locality, horizon and age: Griman Creek Formation, Cenomanian, New South Wales (see Hart et al., 2019: fig. 1).

Diagnosis: Species of *Isisfordia* with the following autapomorphy: caudal maxillary alveoli circular and separated by interalveolar septa along entire caudal portion of the maxillary alveolar groove.

**Remarks**

AM F15818 is affirmed as the holotype specimen of *I. selaslophensis* comb. nov., as its earlier description holds taxonomic seniority. It belongs to *Isisfordia* as it displays the following two apomorphies of the genus, as defined above: caudal maxillary alveolar groove; labiolingually compressed, lingually curved tooth crowns.

**Table:** 

***Isisfordia molnari*Hart et al., 2019**

**Taxonomic assessment**

Holotype: AM F125553 (braincase).

Illustrations of material: Hart et al. (2019: figs. 2, 3).

Locality, horizon and age: Griman Creek Formation, Cenomanian, New South Wales (see Hart et al., 2019: fig. 1).

Diagnosis: Species of *Isisfordia* with the following autapomorphies: flat dorsal surface of the parietal; parietal contribution to medial margin of supratemporal fenestrae flat (does not form raised rim).

**Remarks**

AM F125553 belongs to *Isisfordia* as it displays the following apomorphy of the genus, as defined above: broad exposure of the prootic within the supratemporal foramen rostral to the rostral aperture of the posttemportal canal. Although *I. selaslophensis* comb. nov. and *I. molnari* are morphologically divergent from the type species, *I. duncani*, the former two are based on non-overlapping material. It is therefore likely that *I. molnari* is referable to *I. selaslophensis* comb. nov. and, given the taxonomic seniority of the latter, should be considered a subjective junior synonym of *I. selaslophensis* comb. nov. Definitive assessment of this synonymy is not possible, but should overlapping material be described in the future, this diagnosis can be revised. Although both nomenclatural acts remain valid, there is no compelling evidence at this stage for the presence of two sympatric species of crocodylomorph from the Griman Creek Formation (Hart et al., 2019).

## Conclusions

• *Isisfordia selaslophensis* comb. nov. is instated, with AM F15818 (a partial maxilla) allocated as the type specimen for this species.
• *Isisfordia molnari* remains a valid nomenclatural act, currently represented by AM F125553 (a braincase).
• *Isisfordia molnari* is likely a junior subjective synonym of *I. selaslophensis.* However, as both taxa are currently represented by non-overlapping material, this cannot be determined with certainty.

## Additional Information and Declarations

### Data Availability

The following information was supplied regarding data availability: No raw data or code are associated with this study.

### New Species Registration

The following information was supplied regarding the registration of a newly described species: *Isisfordia selaslophensis* comb. nov. Publication LSID: urn:lsid:zoobank.org:pub:0FD2AD7B-F46D-42C6-B925-D09BACFEFFB0 New combinations do not require an individual ZooBank entry.
